# Supplementary material for: The integrated stress response pathway controls cytokine production in tissue-resident memory CD4+ T cells
Source: Nat Immunol. 2025 Mar 6;26(4):557–66. doi: 10.1038/s41590-025-02105-x (PMC11957990; doi:10.1038/s41590-025-02105-x)
Supplement: Supplementary file 1 — Reporting Summary [file 41590_2025_2105_MOESM1_ESM.pdf]

nature portfolio

Corresponding author(s): Ulf Panzer
Christian Krebs
Hans-Willi Mittrücker

Last updated by author(s): Jan 29, 2025

Reporting Summary

Nature Portfolio wishes to improve the reproducibility of the work that we publish. This form provides structure for consistency and transparency in reporting. For further information on Nature Portfolio policies, see our Editorial Policies and the Editorial Policy Checklist.

Statistics

For all statistical analyses, confirm that the following items are present in the figure legend, table legend, main text, or Methods section.

n/a	Confirmed	
☐	☒	The exact sample size (*n*) for each experimental group/condition, given as a discrete number and unit of measurement
☐	☒	A statement on whether measurements were taken from distinct samples or whether the same sample was measured repeatedly
☐	☒	The statistical test(s) used AND whether they are one- or two-sided<br>*Only common tests should be described solely by name; describe more complex techniques in the Methods section.*
☒	☐	A description of all covariates tested
☐	☒	A description of any assumptions or corrections, such as tests of normality and adjustment for multiple comparisons
☐	☒	A full description of the statistical parameters including central tendency (e.g. means) or other basic estimates (e.g. regression coefficient) AND variation (e.g. standard deviation) or associated estimates of uncertainty (e.g. confidence intervals)
☐	☒	For null hypothesis testing, the test statistic (e.g. *F*, *t*, *r*) with confidence intervals, effect sizes, degrees of freedom and *P* value noted<br>*Give P values as exact values whenever suitable.*
☒	☐	For Bayesian analysis, information on the choice of priors and Markov chain Monte Carlo settings
☒	☐	For hierarchical and complex designs, identification of the appropriate level for tests and full reporting of outcomes
☒	☐	Estimates of effect sizes (e.g. Cohen's *d*, Pearson's *r*), indicating how they were calculated

Our web collection on statistics for biologists contains articles on many of the points above.

Software and code

Policy information about availability of computer code

Data collection	FACS symphony<br>Zeiss LSM800 confocal microscope
Data analysis	FlowJo software 10.5.3<br>GraphPad Prism 8, 10<br>R studio, Jupyter Notebook<br>ZEN software (Carl Zeiss, Jena, Germany)

For manuscripts utilizing custom algorithms or software that are central to the research but not yet described in published literature, software must be made available to editors and reviewers. We strongly encourage code deposition in a community repository (e.g. GitHub). See the Nature Portfolio guidelines for submitting code & software for further information.

Data

Policy information about availability of data

All manuscripts must include a data availability statement. This statement should provide the following information, where applicable:

- Accession codes, unique identifiers, or web links for publicly available datasets
- A description of any restrictions on data availability
- For clinical datasets or third party data, please ensure that the statement adheres to our policy

scRNA-seq data of T cells from human healthy kidney tissue and Trm cell-induced murine kidneys are available at GSE270533 and FigShare webpage (https://figshare.com/s/7912de1afc7fd5bbefd4). The scRNA-seq data of the healthy colon and lung were obtained from GSE157477 and Cross-Tissue Immune Cell Atlas (https://www.tissueimmunecellatlas.org/#publication), respectively. The transcriptome data of European Renal cDNA Bank (kidney biopsy transcriptome datasets) is available from GSE104948 and refine.bio webpage. (https://www.refine.bio/experiments/GSE104948/glomerular-transcriptome-from-european-renal-cdna-bank-subjects-and-living-donors). The transcriptome datasets of colon tissues from Crohn's disease and healthy individuals are available from GSE109142. For the reference genome, refdata-cellranger-hg19-1.2.0 (human) and refdata-gex-mm10-2020-A (mouse) were used.All other data needed to verify the study's conclusions are contained in the manuscript or the Supplementary Materials.

Research involving human participants, their data, or biological material

Policy information about studies with human participants or human data. See also policy information about sex, gender (identity/presentation), and sexual orientation and race, ethnicity and racism.

Reporting on sex and gender	No sex- and gender-based analyses have been performed because of low sample sizes.
Reporting on race, ethnicity, or other socially relevant groupings	not applicable
Population characteristics	No data on population characteristics was collected.
Recruitment	Human kidney tissue was obtained from patients included in the Hamburg GN Registry, the European Renal cDNA Bank, and the Clinical Research Unit 228 (CRU 228) ANCA-GN cohort.
Ethics oversight	The research studies were conducted with the approval of the Ethik-Kommission der Ärztekammer Hamburg (the local ethics committee of the Chamber of Physicians in Hamburg) and in compliance with the ethical principles outlined in the Declaration of Helsinki.

Note that full information on the approval of the study protocol must also be provided in the manuscript.

Field-specific reporting

Please select the one below that is the best fit for your research. If you are not sure, read the appropriate sections before making your selection.

☒ Life sciences ☐ Behavioural & social sciences ☐ Ecological, evolutionary & environmental sciences

For a reference copy of the document with all sections, see nature.com/documents/nr-reporting-summary-flat.pdf

Life sciences study design

All studies must disclose on these points even when the disclosure is negative.

Sample size	Sample sizes were determined based on prior research conducted in our laboratories to use sufficient numbers of mice or cells in each group.
Data exclusions	no data were excluded
Replication	All findings were confirmed twice or more
Randomization	Mice were matched for age and sex before randomization,
Blinding	Investigators were aware of the group allocation because the treatment groups needed to be clear when performing the experiments.

Reporting for specific materials, systems and methods

We require information from authors about some types of materials, experimental systems and methods used in many studies. Here, indicate whether each material, system or method listed is relevant to your study. If you are not sure if a list item applies to your research, read the appropriate section before selecting a response.

Materials & experimental systems

n/a	Involved in the study
☐	☒ Antibodies
☐	☒ Eukaryotic cell lines
☒	☐ Palaeontology and archaeology
☐	☒ Animals and other organisms
☒	☐ Clinical data
☒	☐ Dual use research of concern
☒	☐ Plants

Methods

n/a	Involved in the study
☒	☐ ChIP-seq
☐	☒ Flow cytometry
☒	☐ MRI-based neuroimaging

Antibodies

Antibodies used

human: CD45 (HI30, BioLegend), CD3 (OKT3, BioLegend), CD4 (RPA-T4, BioLegend), CD8 (RPA-T8, BioLegend), CD69 (FN50, BioLegend), CD45RA (HI100, BioLegend), GM-CSF (BVD2-21C11, BioLegend), IL-17A (BL168, BioLegend), IL-17F (SHLR17, eBioscience), IFN-γ (4S.B3, BioLegend), TNF-α (MAB11, BioLegend), CCR7/CD197 (Go43H7, BioLegend);
mouse: CD45 (30-F11, BioLegend), CD3 (145-2C11, BioLegend), CD4 (RM4-5, BioLegend), CD8 (53-6.7, BioLegend), GM-CSF (MP1-22E9, BioLegend), IL-17A (TC11-18H10, BioLegend), IL-17F (9D3.1C8, BioLegend), IL-22 (poly5164, BioLegend), IFN-γ (XMG1.2, BioLegend), TNF-α (MP6-XT22, BioLegend). All antibodies were diluted at a ratio of 1:100 to 1:200.

Validation

Antibodies used in this study are commercially available and have been validated by the manufacturers. Validation statements are provided on the manufacture's website.
human antibody CD45 HI30 BioLegend
https://www.biolegend.com/de-at/products/purified-anti-human-cd45-antibody-710
human antibody CD3 OKT3 BioLegend
https://www.biolegend.com/de-de/products/purified-anti-human-cd3-antibody-3642
human antibody CD4 RPA-T4 BioLegend
https://www.biolegend.com/de-at/products/purified-anti-human-cd4-antibody-830
human antibody CD8 RPA-T8 BioLegend
https://www.biolegend.com/de-de/products/purified-anti-human-cd8a-antibody-839
human antibody CD69 FN50 BioLegend
https://www.biolegend.com/en-ie/products/pe-anti-human-cd69-antibody-1672
human antibody CD45RA HI100 BioLegend
https://www.biolegend.com/nl-be/products/purified-anti-human-cd45ra-antibody-689
human antibody GM-CSF BVD2-21C11 BioLegend
https://www.biolegend.com/en-ie/products/pe-anti-human-gm-csf-antibody-916?GroupID=BLG1622
human antibody IL-17A BL168 BioLegend
https://www.biolegend.com/en-ie/products/purified-anti-human-il-17a-antibody-4442
human antibody IL-17F SHLR17 eBioscience
https://www.thermofisher.com/antibody/product/IL-17F-Antibody-clone-SHLR17-Monoclonal/16-7169-82
human antibody IFN-γ 4S.B3 BioLegend
https://www.biolegend.com/de-de/products/pe-anti-human-ifn-gamma-antibody-1011?GroupID=BLG10006
human antibody TNF-α MAB11 BioLegend
https://www.biolegend.com/ja-jp/products/purified-anti-human-tnf-alpha-antibody-1347
human antibody CCR7/CD197 Go43H7 BioLegend
https://www.biolegend.com/de-at/products/pe-anti-human-cd197-ccr7-antibody-7498
mouse antibody CD45 30-F11 BioLegend
https://www.biolegend.com/nl-be/products/purified-anti-mouse-cd45-antibody-102
mouse antibody CD3 145-2C11 BioLegend
https://www.biolegend.com/fr-lu/products/apc-anti-mouse-cd3epsilon-antibody-21
mouse antibody CD4 RM4-5 BioLegend
https://www.biolegend.com/nl-nl/products/purified-anti-mouse-cd4-antibody-484
mouse antibody CD8 53-6.7 BioLegend
https://www.biolegend.com/nl-be/products/apc-anti-mouse-cd8a-antibody-150
mouse antibody GM-CSF MP1-22E9 BioLegend
https://www.biolegend.com/ja-jp/products/pe-anti-mouse-gm-csf-antibody-958
mouse antibody IL-17A TC11-18H10 BioLegend
https://www.biolegend.com/nl-be/products/purified-anti-mouse-il-17a-antibody-1634
mouse antibody IL-17F 9D3.1C8 BioLegend
https://www.biolegend.com/fr-ch/products/pe-anti-mouse-il-17f-antibody-6964
mouse antibody IL-22 poly5164 BioLegend
https://www.biolegend.com/nl-be/products/pe-anti-mouse-il-22-antibody-6486
mouse antibody IFN-γ XMG1.2 BioLegend
https://www.biolegend.com/fr-ch/products/purified-anti-mouse-ifn-gamma-antibody-998
mouse antibody TNF-α MP6-XT22 BioLegend
https://www.biolegend.com/de-de/products/pe-anti-mouse-tnf-alpha-antibody-978?GroupID=GROUP24

Eukaryotic cell lines

Policy information about cell lines and Sex and Gender in Research

Cell line source(s)	HEK293T (https://www.atcc.org/products/crl-3216)
Authentication	not authenticated
Mycoplasma contamination	Cells which tested negative for mycoplasma were used for experiments
Commonly misidentified lines (See ICLAC register)	No commonly misidentified cell lines were used.

Animals and other research organisms

Policy information about studies involving animals; ARRIVE guidelines recommended for reporting animal research, and Sex and Gender in Research

Laboratory animals	Male mice on a C57BL/6 background, aged between 8 and 16 weeks, were used. Mice were maintained in specific pathogen-free conditions with controlled humidity and temperature with a light/dark cycle of 12h each.
Wild animals	not used
Reporting on sex	only males were used
Field-collected samples	No field-collected samples were used in this study.
Ethics oversight	Experiments with mice followed the national guidelines, and local ethics committees (Behörde für Justiz und Verbraucherschutz Hamburg) approved the research protocols.

Note that full information on the approval of the study protocol must also be provided in the manuscript.

Plants

Seed stocks	n.a.
Novel plant genotypes	n.a.
Authentication	n.a.

Flow Cytometry

Plots

Confirm that:

☒ The axis labels state the marker and fluorochrome used (e.g. CD4-FITC).

☒ The axis scales are clearly visible. Include numbers along axes only for bottom left plot of group (a 'group' is an analysis of identical markers).

☒ All plots are contour plots with outliers or pseudocolor plots.

☒ A numerical value for number of cells or percentage (with statistics) is provided.

Methodology

Sample preparation	Single-cell suspensions were obtained from kidney and blood samples to isolate and analyze human leukocytes. Kidney tissue was enzymatically digested with collagenase D at 0.4 mg/ml (Roche, Mannheim, Germany) and DNase I (10 µg/ml, Sigma-Aldrich, Saint Louis, MO) in RPMI 1640 medium at 37° C for 30 minutes, followed by dissociation with gentleMACS (Miltenyi Biotec). Blood samples were separated using Leucosep tubes (Greiner Bio-One, Kremsmünster, Austria). Samples were filtered through a 30-µm filter (Partec, Görlitz, Germany) prior to antibody staining and flow cytometry.<br>Cells from murine spleens were isolated by squashing the organ through a 70-µm cell strainer. Erythrocytes were lysed using a lysis buffer (155 mM NH4Cl, 10 mM KHCO3, 10 µM EDTA, pH 7.2). To isolate renal lymphocytes from mice, kidneys were enzymatically digested with 400 µg/ml collagenase D (Roche) and 10 U/ml DNase I (Sigma-Aldrich) at 37°C for 30 min.

Subsequently, leukocytes were isolated by density gradient centrifugation using 37% Easycoll (Merck Millipore) and a filtration step using a 30-µm cell strainer (Partec). T cell isolation from the intestine is described previously. Briefly, murine intestine was cut longitudinally after removing Peyer's patches and adventitial fat. To collect intraepithelial lymphocytes, the intestine tissue was incubated in HBSS containing 1mM dithioerythritol followed by a dissociation step using 1 mM EDTA for 20 min at 37° C. To collect lamina propria lymphocytes, the tissue was cut into small pieces and incubated for 45 min at 37° C in HBSS supplied with 1 mg/ml collagenase and 10 U/ml DNase I. Leukocytes were further enriched by Percoll gradient. Intraepithelial cells and lamina propria lymphocytes were pooled for analysis.

Instrument

FACS symphony

Software

FlowJo

Cell population abundance

Cell populations were abundant enough for any of the analysis. Over 5000 cells of each target cell population were detected from the kidney and intestine of human and mice.

Gating strategy

The initial gate on FSC/SSC plots was set to remove cell debris and single cells were gated according to FSC-W and FSC-H. After gating on live cells, target cell populations were gated. Trm cells were gated as CD45+ CD4+ CD44+ CD69+ CD62L-; Tem cells as CD45+ CD4+ CD44+ CD69- CD62L-; Tcm cells as CD45+ CD4+ CD44+ CD69- CD62L+; Naïve T cells as CD45+ CD4+ CD44- CD69- CD62L+.

☒ Tick this box to confirm that a figure exemplifying the gating strategy is provided in the Supplementary Information.
